# The cost-effectiveness of transcatheter aortic valve implantation: exploring the Italian National Health System perspective and different patient risk groups

**DOI:** 10.1007/s10198-021-01314-z

**Published:** 2021-05-21

**Authors:** V. Lorenzoni, G. Barbieri, F. Saia, F. Meucci, G. L. Martinelli, A. G. Cerillo, S. Berti, P. Candolfi, G. Turchetti

**Affiliations:** 1 Institute of Management, Scuola Superiore Sant’Anna, Pisa, Italy; 2 Edwards Lifesciences Italia S.p.A, Milan, Italy; 3 Cardio-Thoracic-Vascular Department, RCCS University Hospital of Bologna, Policlinico S. Orsola, Bologna, Italy; 4 Division of Cardiac Surgery, Careggi University Hospital, Florence, Italy; 5 Department of Cardiac Surgery, IRCCS MultiMedica Sesto San Giovanni, Milano, Italy; 6 Fondazione C.N.R Regione Toscana G. Monasterio, Massa, Italy; 7 Edwards Lifesciences S.A., Nyon, Switzerland; 8 Azienda Ospedaliero Universitaria Careggi, Florence, Italy

**Keywords:** Aortic stenosis, Economic, Cost-effectiveness, Transcatheter aortic valve implantation, I12, I18

## Abstract

**Objectives:**

To assess the cost-effectiveness (CE) of transcatheter aortic valve implantation (TAVI) in Italy, considering patient groups with different surgical risk.

**Methods:**

A Markov model with a 1-month cycle length, comprising eight different health states, defined by the New York Heart Association functional classes (NYHA I–IV), with and without stroke plus death, was used to estimate the CE of TAVI for intermediate-, high-risk and inoperable patients considering surgical aortic valve replacement or medical treatment as comparators according to the patient group. The Italian National Health System perspective and 15-year time horizon were considered. In the base-case analysis, effectiveness data were retrieved from published efficacy data and total direct costs (euros) were estimated from national tariffs. A scenario analysis considering a micro-costing approach to estimate procedural costs was also considered. The incremental cost-effectiveness ratio (ICER) was expressed both in terms of costs per life years gained (LYG) and costs per quality adjusted life years (QALY). All outcomes and costs were discounted at 3% per annum. Univariate and probabilistic sensitivity analyses (PSA) were performed to assess robustness of results.

**Results:**

Over a 15-year time horizon, the higher acquisition costs for TAVI were partially offset in all risk groups because of its effectiveness and safety profile. ICERs were €8338/QALY, €11,209/QALY and €10,133/QALY, respectively, for intermediate-, high-risk and inoperable patients. ICER values were slightly higher in the scenario analysis. PSA suggested consistency of results.

**Conclusions:**

TAVI would be considered cost-effective at frequently cited willingness-to-pay thresholds; further studies could clarify the CE of TAVI in real-life scenarios.

**Supplementary Information:**

The online version contains supplementary material available at 10.1007/s10198-021-01314-z.

## Introduction

Aortic valve disease represents a relevant public health problem, whose prevalence is expected to increase with population ageing [1, 2]. Currently available options for the treatment of aortic stenosis (AS) include surgical valve replacement (sAVR), transcatheter aortic valve implantation (TAVI) and medical treatment. The updated European guidelines for the management of AS recommend that the choice of appropriate treatment be determined by the heart team on the basis of individual patient characteristics, surgical risks, anatomic and technical considerations; generally, the use of TAVI is recommend for patients not suitable for sAVR and elderly patients with increased surgical risk (Society of Thoracic Surgeon [STS] score or EuroSCORE II ≥ 4%) [3–5].

On the basis of population trend and current indication of TAVI, a recent study estimated the annual number of TAVI candidates was approximately 115,000 in Europe, with less than 16,000 in Italy [6]. These figures increased considerably when considering the extension of indication for TAVI also to lower risk groups [6], according to available clinical evidence [7–9].

The estimated requirements for TAVI, coupled with actual volume of procedures performed [10], opens challenges related to both feasibility—given the current extensive expertise and infrastructural equipment of centres—and sustainability for the health care systems in the near future.

The existing health economic evaluations of TAVI, mainly refer to previous generation valves and are based on the early trials, suggesting a different probability of TAVI being cost-effective depending on the alternatives considered and also on the context explored [11–18]. Indeed, from the very first-generation valve marketed in 2007, devices have been iteratively developed and improved. Experience with the procedure has progressed over time and changes in the management of patients undergoing TAVI have occurred in contemporary clinical practice. As a result, despite limited research, the most recent studies on the clinical and economic impact of TAVI suggested that device modification affected clinical and economic outcomes (i.e., a lower delivery profile and an external sealing skirt to reduce paravalvular regurgitation) and accumulation of evidence about contemporary devices is essential to inform current decision-making and practices [19].

The issue is indeed challenging in many European countries and in Italy, where, similarly to other innovative health technologies, the affordability of TAVI represents an unanswered question, given the acquisition cost of devices involved, the lack of evidence in the specific context and the current absence of specific reimbursement for that procedure that could be used all over the region of the country. With this scenario, despite the promising clinical results in many risk groups and the projection for TAVI requirement, the actual prevalence of the procedure is undermined by organizational and regulatory issues.

Accordingly, the aim of the present study is to assess the cost-effectiveness (CE) of TAVI considering the last generation valve, SAPIEN 3^®^, the perspective of the Italian National Health System (INHS) and patient groups with different surgical risk.

The main objective of the study is to provide evidence to guide decision making in the national context, considering the most updated evidence on clinical effectiveness coming from randomized controlled trials, and exploring different scenarios developed, estimating costs both on the basis of national tariffs but also considering a micro-costing approach to the value of procedures.

## Methods

### Analytical framework

A CE analysis was performed to evaluate the health economics implications related to the use of TAVI for the treatment of AS. The base-case analysis considered the perspective of the INHS and a 15-year time horizon. The analysis was performed adapting to the Italian context a Markov model developed as a Microsoft Excel^®^ macro-enabled workbook to evaluate the incremental cost-effectiveness ratio (ICER) of TAVI versus alternative approaches. The results are presented both in terms of incremental costs per life years gained (LYG) and as incremental costs per quality adjusted life years (QALY).

Depending on the risk of survival after surgical intervention (as commonly defined on the basis of the STS risk score [4] or the EUROSCORE [5] and the heart team evaluation), the model allows evaluation of three different patient populations—intermediate-risk, high-risk and inoperable patients—and comparison with different treatment standards: sAVR for intermediate- and high-risk patients and medical treatment for inoperable patients.

**Fig. 1:**
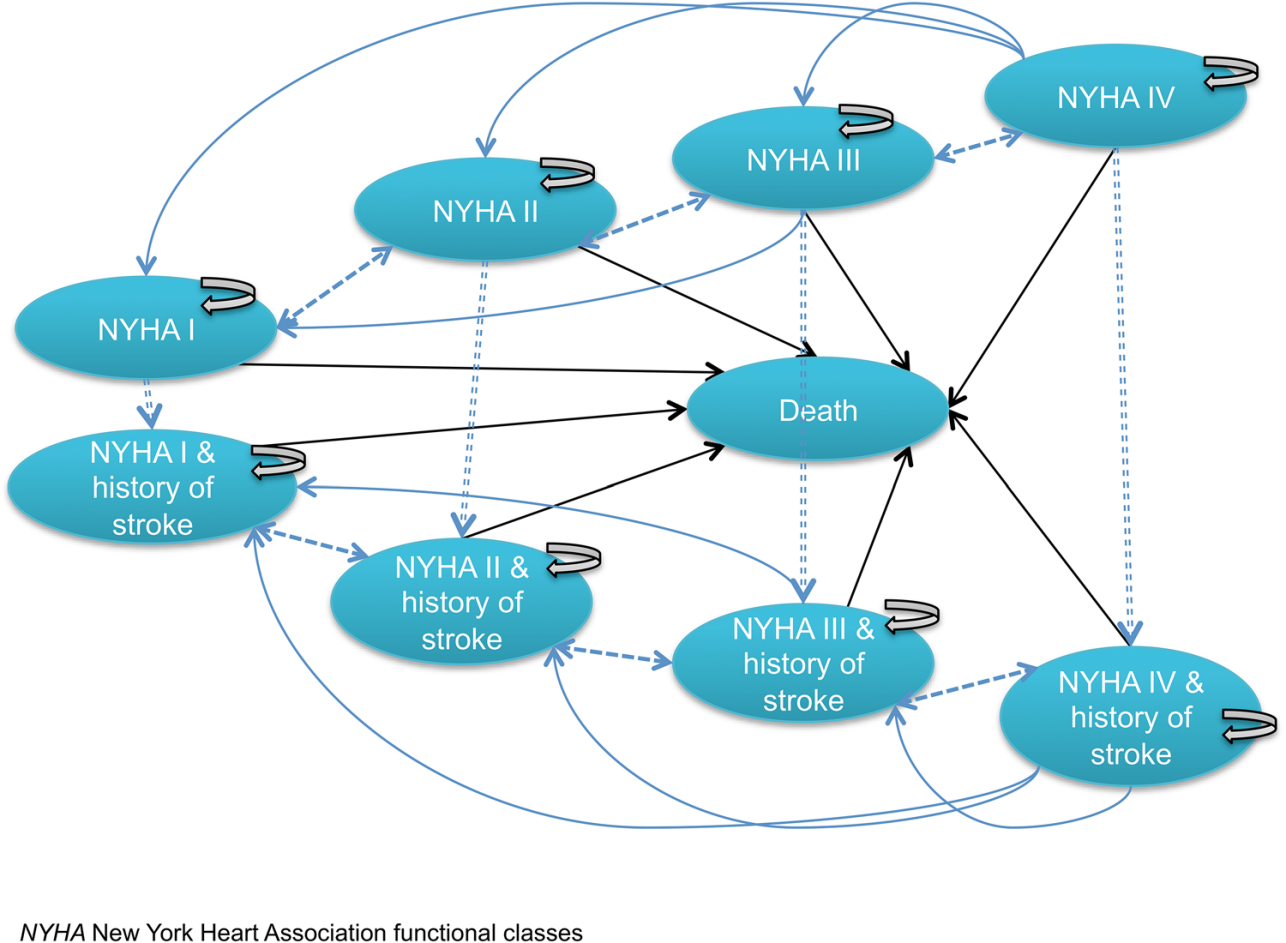
Structure of the Markov model used for the analysis

The structure of the Markov model used in the analysis was common to all risk groups; in particular, the model considered 1-month cycle length and eight different health states, defined by New York Heart Association functional classes (NYHA I–IV) with and without history of stroke, plus death (Fig. 1).

Patients transitioned between health states according to the progression or improvement of the disease over the four severity levels of NYHA classes and even they are allowed to experience procedural complications as well as relevant clinical events according to Valve Academic Research Consortium (VARC) 2 criteria [20] over the follow-up.

### Clinical data

Transition probabilities (related to disease progression or improvement), risk of relevant clinical events and survival were derived from the Placement of Aortic Transcatheter Valves (PARTNER) trials [7, 21–23] and extrapolation from them all over the time horizon of the analysis as previously detailed in Goodall et al. [24]; particularly, linear extrapolation was used to extend mortality data, while for complications, the last available data were assumed constant for the rest or the time period. Details of inputs for the incidence of events used in the analysis over the different risk groups and references for them are reported in Table 1. Details about the data used to derive transition probabilities are showed in Table A1.

**Table 1:** Details of inputs data used in the base-case analysis to model the risk of relevant clinical events

	Intermediate risk		High risk		Inoperable	
	TAVI^a^	sAVR^b^	TAVI^c^	sAVR^d^	TAVI	Medical treatment
Mortality						
1 month	1.0%	3.5%	3.1%	5.7%	2.0%	6.1%
2–6 months	3.9%	4.8%	3.9%	7.2%	8.5%	23.5%
7 months–1 year	4.1%	5.1%	4.8%	8.7%	9.4%	25.6%
13 months–2 years	7.6%	8.0%	16.7%	16.7%	24.5%	42.9%
25 months–3 years	8.3%	8.7%	17.1%	17.1%	25.3%	41.5%
37 months–4 years	9.0%	9.5%	17.6%	17.6%	25.8%	40.7%
49 months–5 years	9.9%	10.5%	18.1%	18.1%	26.0%	40.4%
Major stroke						
1 month	1.1%	4.5%	1.3%	1.3%	0.0%	1.1%
2–6 months	0.9%	0.8%	0.8%	0.0%	0.0%	1.7%
7 months –1 year	0.5%	0.8%	0.9%	0.4%	1.2%	0.8%
13 months–2 years	0.5%	0.9%	0.9%	1.3%	1.2%	0.0%
Transient ischaemic attack						
1 month	0.5%	0.4%	0.8%	0.3%	0.5%	0.0%
2–6 months	0.8%	0.7%	0.5%	0.7%	0.0%	0.0%
7 months–1 year	0.8%	0.8%	1.4%	0.4%	0.0%	0.0%
13 months–2 years	0.8%	0.5%	1.4%	1.3%	0.0%	0.0%
Atrial fibrillation						
1 month	5.5%	28.1%	6.0%	18.2%	2.5%	1.1%
2–6 months	0.3%	0.6%	1.9%	1.1%	1.0%	4.1%
7 months–1 year	0.7%	0.2%	1.4%	0.4%	0.0%	1.7%
13 months–2 years	0.7%	0.1%	1.4%	0.0%	0.0%	0.0%
Renal replacement therapy						
1 month	0.5%	3.3%	1.0%	4.5%	1.0%	1.7%
2–6 months	0.0%	1.8%	0.0%	1.1%	0.0%	1.7%
7 months–1 year	0.0%	0.5%	0.0%	0.8%	0.0%	0.8%
13 months–2 years	0.0%	1.1%	0.0%	0.4%	0.0%	3.5%
Myocardial infarction						
1 month	0.3%	1.9%	0.5%	0.3%	0.5%	0.0%
2–6 months	0.0%	0.8%	0.8%	0.0%	1.5%	0.6%
7 months–1 year	0.0%	0.8%	0.8%	0.0%	0.6%	0.0%
13 months–2 years	0.0%	1.1%	0.8%	0.9%	0.6%	1.2%
New pacemaker						
1 month	10.1%	7.2%	10.9%	4.5%	17.6%	5.0%
2–6 months	0.5%	0.9%	1.9%	0.7%	3.1%	2.3%
7 months–1 year	0.1%	1.1%	0.3%	0.0%	0.6%	0.8%
13 months–2 years	0.1%	1.4%	0.3%	1.3%	0.6%	0.0%
Major bleeding						
1 month	10.7%	32.7%	13.8%	24.6%	15.1%	3.9%
2–6 months	0.5%	3.1%	2.7%	2.5%	4.1%	5.2%
7 months–1 year	0.0%	2.7%	1.1%	2.9%	1.7%	5.0%
13 months–2 years	0.0%	2.8%	1.1%	3.5%	1.7%	5.9%
Major vascular complications						
1 month	6.4%	5.8%	4.4%	4.2%	6.5%	1.1%
2–6 months	0.0%	0.3%	0.5%	0.0%	0.0%	0.6%
7 months–1 year	0.0%	0.1%	0.3%	0.0%	0.0%	0.8%
13 months–2 years	0.0%	0.1%	0.3%	0.0%	0.0%	0.0%
Hospitalization for AS symptoms or procedure-related complications						
1 month	4.9%	7.3%	7.0%	5.8%	11.1%	10.1%
2–6 months	4.5%	8.5%	8.3%	9.5%	11.2%	35.8%
7 months–1 year	3.9%	2.7%	4.0%	4.1%	1.2%	26.5%
13 months–2 years	3.9%	3.9%	4.0%	7.4%	1.2%	47.1%
Hospitalization rate for heart failure						
1 month	1.8%	0.0%	3.1%	5.1%	8.0%	7.8%
2–6 months	2.5%	0.0%	5.4%	7.8%	8.2%	31.2%
7 months–1 year	2.9%	0.0%	2.8%	2.9%	0.6%	24.8%
13 months–2 years	2.9%	0.0%	2.8%	4.8%	0.6%	41.2%
Balloon valvuloplasty						
1 month	0.1%	0.0%	0.0%	0.0%	0.0%	67.0%
2–6 months	0.1%	0.0%	0.0%	0.0%	0.0%	13.3%
7 months–1 year	0.0%	0.0%	0.0%	0.0%	0.0%	24.8%
13 months–2 years	0.0%	0.0%	0.0%	0.0%	0.0%	16.5%
(Re-)TAVI						
1 month	0.0%	0.0%	0.0%	0.0%	0.0%	0.0%
2–6 months	0.0%	0.0%	0.3%	0.4%	0.5%	0.6%
7 months–1 year	0.3%	0.0%	0.0%	0.0%	0.0%	0.8%
13 months–2 years	0.3%	0.0%	0.0%	0.0%	0.0%	2.4%
(Re)sAVR						
1 month	0.0%	0.0%	0.0%	0.0%	0.0%	2.2%
2–6 months	0.3%	0.0%	0.0%	0.0%	0.0%	1.7%
7 months–1 year	0.0%	0.5%	0.6%	0.0%	0.0%	2.5%
13 months–2 years	0.0%	0.0%	0.6%	0.0%	0.0%	1.2%
Endocarditis						
1 month	0.2%	0.0%	0.3%	0.3%	0.0%	0.0%
2–6 months	0.3%	0.3%	0.3%	0.7%	0.0%	0.6%
7 months–1 year	0.3%	0.0%	0.9%	0.0%	0.0%	0.0%
13 months–2 years	0.3%	0.1%	0.9%	0.0%	0.0%	0.0%

For the sake of simplicity data over 5-year time horizon were reported for mortality and 2-year time horizon was used for the other inputs

*AF* atrial fibrillation; *DRG* diagnosis-related groups; *HF* heart failure; *sAVR* surgical valve replacement; *TIA* transient ischaemic attack; *TAVI* transcatheter aortic valve implantation

^a^Thournai et al. [21]; ^b^Leon et al. [7]; ^c^Hermann et al. [22]; ^d^Mack et al. [20]; Hermann et al. [22]; Leon et al.[21]

In summary, survival, transition probabilities and the incidence of relevant clinical events among the intermediate-risk group were drawn from Thourani et al. [22] and Leon et al. [7] for TAVI and sAVR, respectively; similarly, the data reported in Herrmann et al. [23] and Mack et al. [21] for TAVI and sAVR, respectively, were used for high-risk patients. For inoperable, survival and the incidence of relevant clinical events were derived from Herrmann et al. [23] and Leon et al. [25].

### Quality adjusted life years

For the diverse risk groups, utilities related to alternative treatment were obtained from published data [26, 27]. Quality adjusted life years (QALY) for the different risk groups all over the time horizon of the analysis were thus estimated according to time spent in the different health states. Health utility value per health state is presented in Table A2.

**Table 2:** Details of costs inputs data used in the base-case analysis

	Costs (euro)	References
TAVI procedure	30,634	TAVI tariff for Emilia Romagna Region [29]
sAVR procedure	24,675	National tariff for DRG105 [27]
Balloon valvuloplasty	3962	National tariff for DRG518 [27]
Pacemaker implantation	4756	National tariff for DRG552 [27]
Stroke	19,624	National tariff for DRG14 [27] + Piscitelli et al.[30]
TIA	2967	National tariff for DRG15 [27]
Major bleeding	3891	National tariff for DRG14 [27]
Major cardiovascular complication	3392	National tariff for DRG124 [27]
HF hospitalization	3051	National tariff for DRG127 [27]
Acute pulmonary oedema	3802	National tariff for DRG87 [27]
Acute onset AF	1090	National tariff for DRG131 [27]
AS hospitalization	6876	National tariff for DRG120 [27]
Renal failure requiring replacement therapy	1381	National tariff for DRG137 [27]
Myocardial infarction	8353	Weighted mean of national tariff for DRG121,122,123 [27, 28]
Endocarditis	10,573	National tariff for DRG126 [27]

*AF* atrial fibrillation; *DRG* diagnosis-related groups; *HF* heart failure; *sAVR* surgical valve replacement; *TIA* transient ischaemic attack; *TAVI* transcatheter aortic valve implantation

### Costs

In the base-case analysis, direct health costs included in the analyses comprised costs related to treatment strategy (i.e., intervention or medical treatment) and costs associated with the management of events all over the time horizon. All costs in the base-case analysis were derived from national tariffs [28, 29]. As in Italy, there is currently no specific national tariff set for TAVI and procedures are reimbursed using tariffs related to the conventional surgical approach; to value the cost of TAVI and in particular to account for the higher costs generally implied by that procedure, the specific tariff set for the Emilia Romagna region in 2014 was used in the analysis [30].

The data from literature were also used to complement information not available from the national tariffs, such as the costs of rehabilitation after stroke or myocardial infarction [31]. Details of costs considered in the analysis are reported in Table 2.

All costs were given in euros and provided up to 2019, except for tariffs that refer to the last year available; outcomes and costs were discounted at 3% per annum.

### Scenario analyses

A scenario analysis considering a micro-costing approach to estimate costs associated with the index intervention was developed. This scenario was intended to fill the gap of the lack of specific tariff related to TAVI in Italy and to provide an alternative picture of costs associated with treatments. The micro-costing analysis involved constructing, in each centre involved, the model of a “typical” procedure of sAVR and TAVI, then estimating costs starting with main resources used and related unit prices. The model for costs was based on the internal hospital protocols and feedback from expert clinicians involved.

In detail, four Italian centres experienced in the treatment of AS with TAVI were contacted through reference clinicians and an ad hoc format requesting both TAVI and sAVR procedures, information related to quantity and type of personnel involved, consumables, operating time (skin-to-skin), intensive and sub-intensive unit stay and general ward stay.

Unit costs were obtained from one of the centres involved and representativeness of costs collected in the other centres was checked with reference clinicians from the other centres. The hospital administration provided data on the unit cost per hour (including overhead) for the services of staff members, such as surgeons, anaesthesiologists and nurses. The cost of labour was then calculated as the product of the cost per minute by the operating time (in minutes). The administrative offices of the hospital provided a detailed accounting of consumable costs per procedure.

Costs associated with “typical” TAVI and sAVR procedures were thus obtained multiplying unit costs for the weighted average of resource used in the different centres considering the number of procedures performed in each centre as weight. All costs were adjusted to the 2019 value of the euro.

Direct health costs related to rehabilitation after TAVI or sAVR procedures were also included in the scenario analysis, to fully capture costs related to the different approaches.

In outline, costs of rehabilitation were valued considering costs of rehabilitation after hospital discharge according to the Tariff List for rehabilitation and long-term stay elaborated in 2013 by the Ministry of Health [28] and length of rehabilitation from a previous study on real-world data in Italy [32]. On the basis of these data, 64% and 6.2% of patients undergoing sAVR and TAVI, respectively, were considered to require rehabilitation after the procedure and associated costs were valued considering national tariffs related to rehabilitation or long-term care admissions and in particular using Diagnosis Related Groups (DRG) related to Major Diagnostic Category 5 (circulatory system diseases and disorders).

### Sensitivity analysis

To evaluate robustness of the results both one-way (OWSA) and probabilistic sensitivity analyses were conducted.

An one-way sensitivity analyses were conducted on all model parameters associated with uncertainty modifying base-case inputs by ± 20% and showing main results from the analysis in Tornado diagrams showing the twenty main drivers of the analyses.

In PSA, appropriate statistical distributions were assigned to input values used in the analysis and 1000 simulation runs were used drawing samples from those distributions. In detail, Normal distribution was used for costs valued on the basis of tariff while Beta distributions were assumed for both the incidence of events and utilities. The results from the PSA are represented on the CE plane and CE acceptability curves were also derived to represent decision uncertainty in terms of probability that TAVI is cost-effective conditional on a range of possible CE thresholds.

## Results

**Fig. 2:**
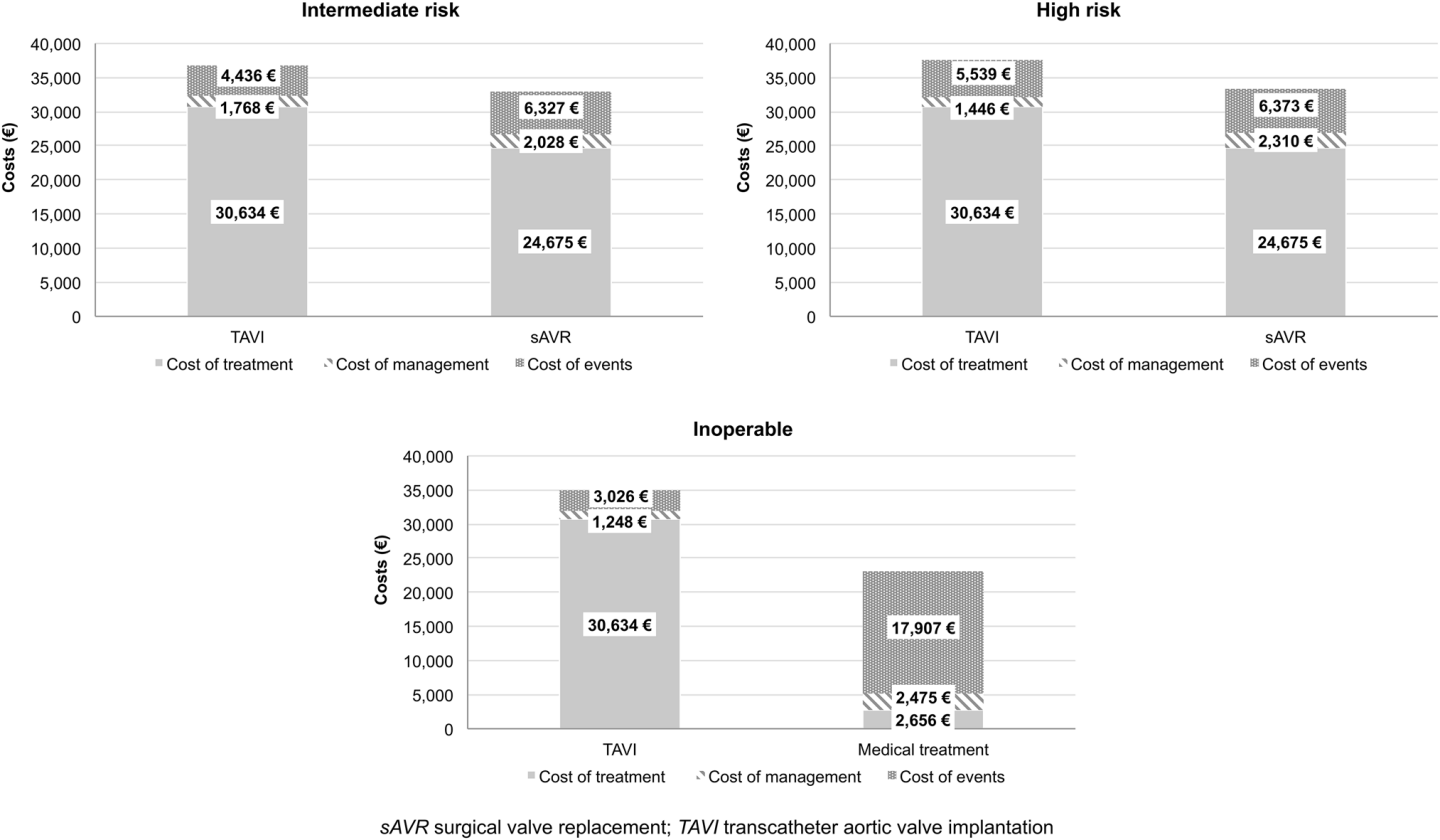
Costs of alternatives over the 15-year time horizon by costs item

In the base-case analysis, QALY and LYG were higher for TAVI versus alternatives in all risk groups. In particular, costs for the indexed hospitalization were higher in all risk groups and were partly offset by costs of patient management and follow-up events (Fig. 2).

The results from the CE analysis suggested that additional costs for TAVI increased according to patient risk ranging from less than €4000/patient to approximately €12,000/patient, for intermediate-risk and inoperable groups, respectively. TAVI also resulted in additional QALY and LYG, which were higher in inoperable groups (Table 3).

**Table 3:** Results from the base-case cost-effectiveness analysis over a 15-year time horizon

	Overall costs	QALY	LY	Δ costs (€)	Δ QALY	Δ LY	ICUR	ICER
Intermediate risk								
TAVI	36,623	4.21	6.08	3593	0.43	0.45	8338	8035
sAVR	33,030	3.78	5.64					
High risk								
TAVI	37,189	2.83	4.49	3831	0.34	0.40	11,209	9474
sAVR	33,358	2.49	4.08					
Inoperable								
TAVI	34,908	1.83	3.17	11,920	1.18	1.57	10,133	7577
Medical treatment	22,989	0.65	1.60					

*ICER* incremental cost-effectiveness ratio; *ICUR* incremental cost–utility ratio; *LYG* life years gained; *QALY* quality adjusted life years; *sAVR* surgical valve replacement; *TAVI* transcatheter aortic valve implantation

Indeed, incremental cost–utility ratio (ICUR) values were €8338/QALY among intermediate-risk patients, €11,209/QALY in the high-risk patients and €10,133/QALY among the inoperable group. Similarly, ICER were €8035/LYG, €9474/LYG and €7577/LYG, respectively.

### Scenario analysis

Micro-costing estimate costs for the indexed procedure were €26,985 for TAVI and €14,802 for sAVR. When assuming those costs value for the indexed intervention and also including direct health costs for rehabilitation, both ICERs and ICURs were higher as compared to the base-case analysis and remained below the conventional thresholds.

In this scenario, increasing additional costs for the indexed TAVI procedure over a 15-year time horizon was less than €10,000 in all risk groups. This resulted in incremental costs per patient being €7783/QALY and €5820/LYG among inoperable groups (Table 4). Among intermediate- and high-risk patients, the gain induced by TAVI in terms of both QALYs and LYG was lower as compared to inoperable; ICUR was comprised between €16,771/QALY and €21,417/QALY, respectively, while ICER values resulted in €16,161/LYG and €18,101/LYG, respectively.

**Table 4:** Scenario analysis: results of the cost-effectiveness analysis over 15 year

	Overall costs	QALY	LY	Δ costs (€)	Δ QALY	Δ LY	ICUR	ICER
Intermediate risk								
TAVI	33,161	4.21	6.08	7227	0.43	0.45	16,771	16,161
sAVR	25,935	3.78	5.64					
High risk								
TAVI	33,551	2.83	4.49	7319	0.34	0.40	21,417	18,101
sAVR	26,232	2.49	4.08					
Inoperable								
TAVI	31,517	1.83	3.17	9155	1.18	1.57	7783	5820
Medical treatment	22,362	0.65	1.60					

*ICER* incremental cost-effectiveness ratio; *ICUR* incremental cost–utility ratio; *LYG* life years gained; *QALY* quality adjusted life years; *sAVR* surgical valve replacement; *TAVI* transcatheter aortic valve implantation

Conclusions were similar when considering micro-costing to estimate the value of the diverse procedures, but not including rehabilitation costs in the analysis (data not shown).

### Sensitivity analyses

**Fig. 3:**
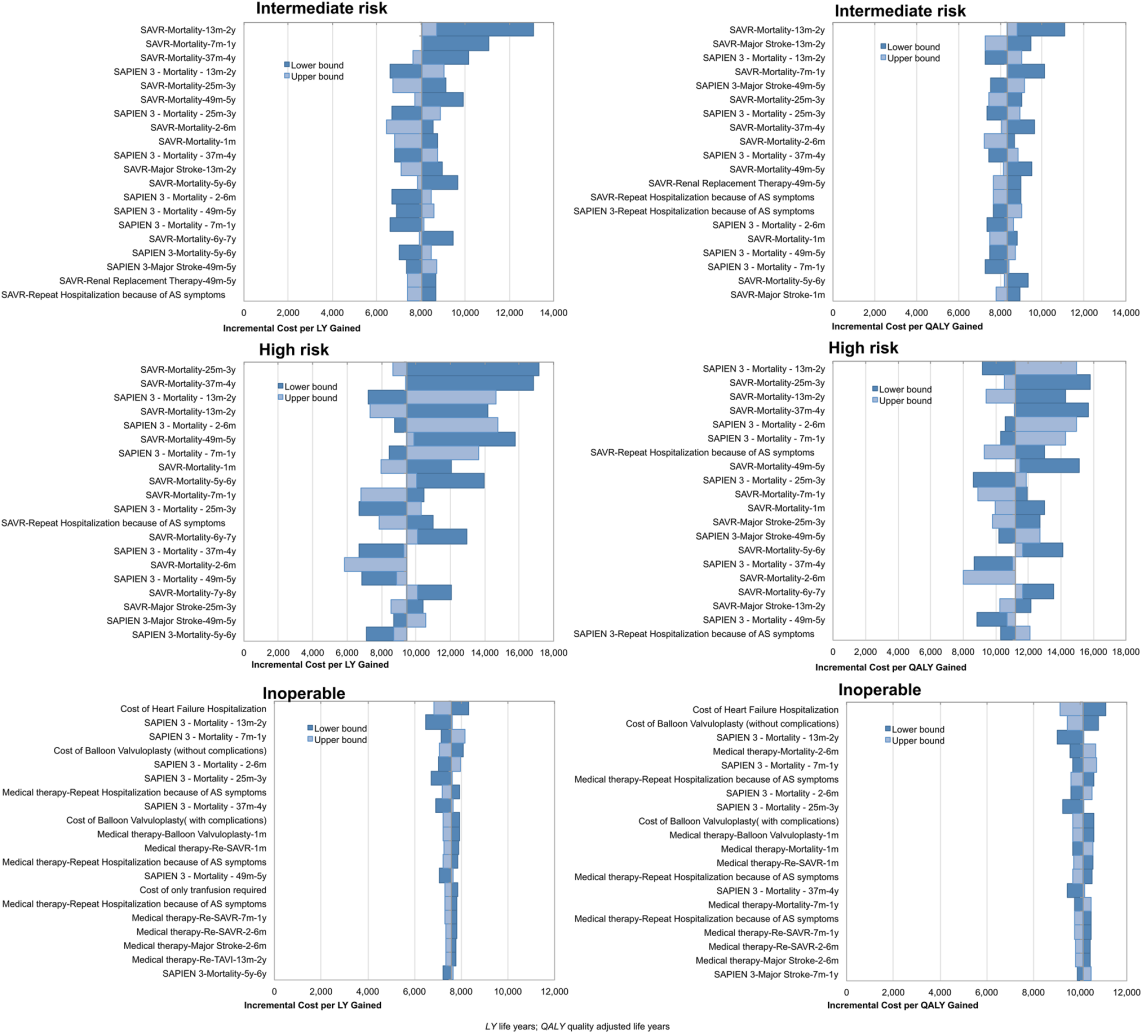
Tornado diagrams showing main results from the one-way sensitivity analysis

Tornado diagrams displaying the results from OWSA are shown in Fig. 3, all over the analyses performed OWSA suggested that mortality along the time horizon was one of the main driver of the analyses particularly for intermediate and high risk groups. In those groups major incidence of stroke in the short term and repeated hospitalizations for AS were also among the main driver of base-case results. Among inoperable, the results from the base-case analyses were mainly influenced by mortality, re-operation risk for sAVR, risk and costs of hospitalizations for HF and valvuloplasty.

**Fig. 4:**
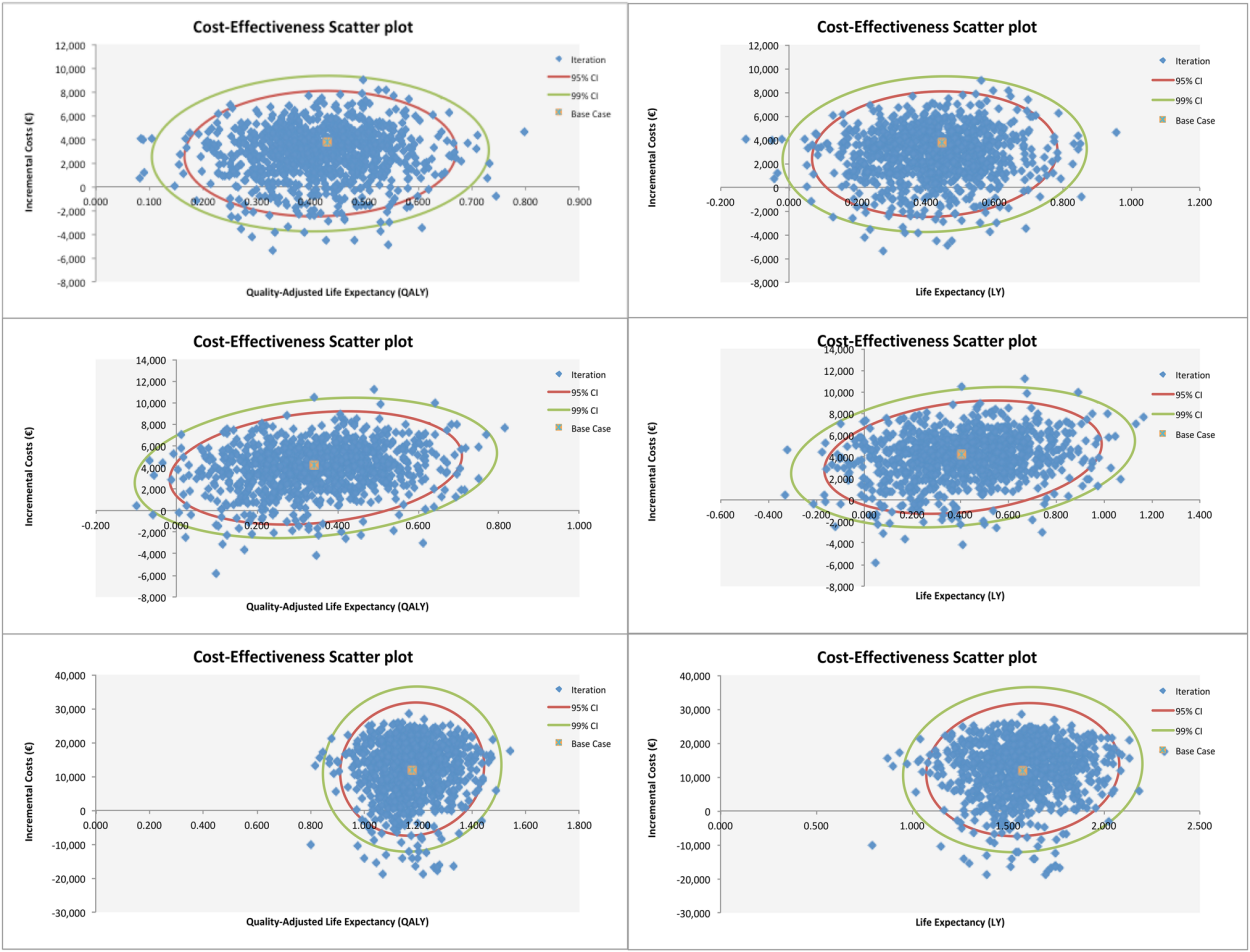
Results from the probabilistic sensitivity analysis

Scatterplots of willingness-to-pay per LYG and per QALY gained resulting from the PSA suggested consistency of results from the base-case analysis as shown in Fig. 4.

**Fig. 5:**
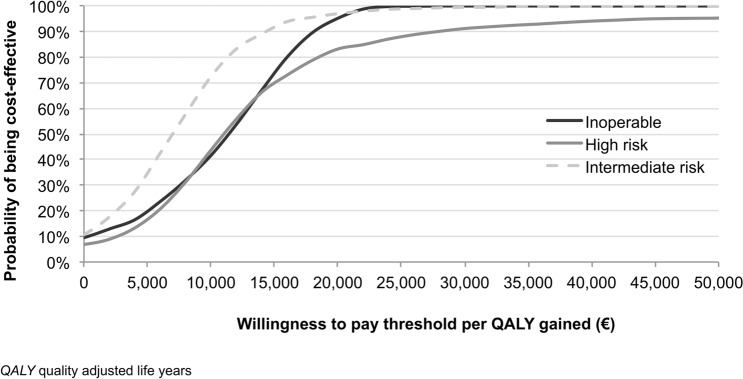
Cost-effectiveness acceptability curves for TAVI over different risk groups

Cost-effectiveness acceptability curves, shown in Fig. 5, suggest that considering conventional thresholds defined at national level (typically comprised between €25,000–40,000/QALY [33, 34]), TAVI showed high probability (in the range of about 90–100%) of being cost-effective in all risk groups, both when considering the upper and lower limits of the range of value generally considered in Italy (Fig. 5).

**Fig. 6:**
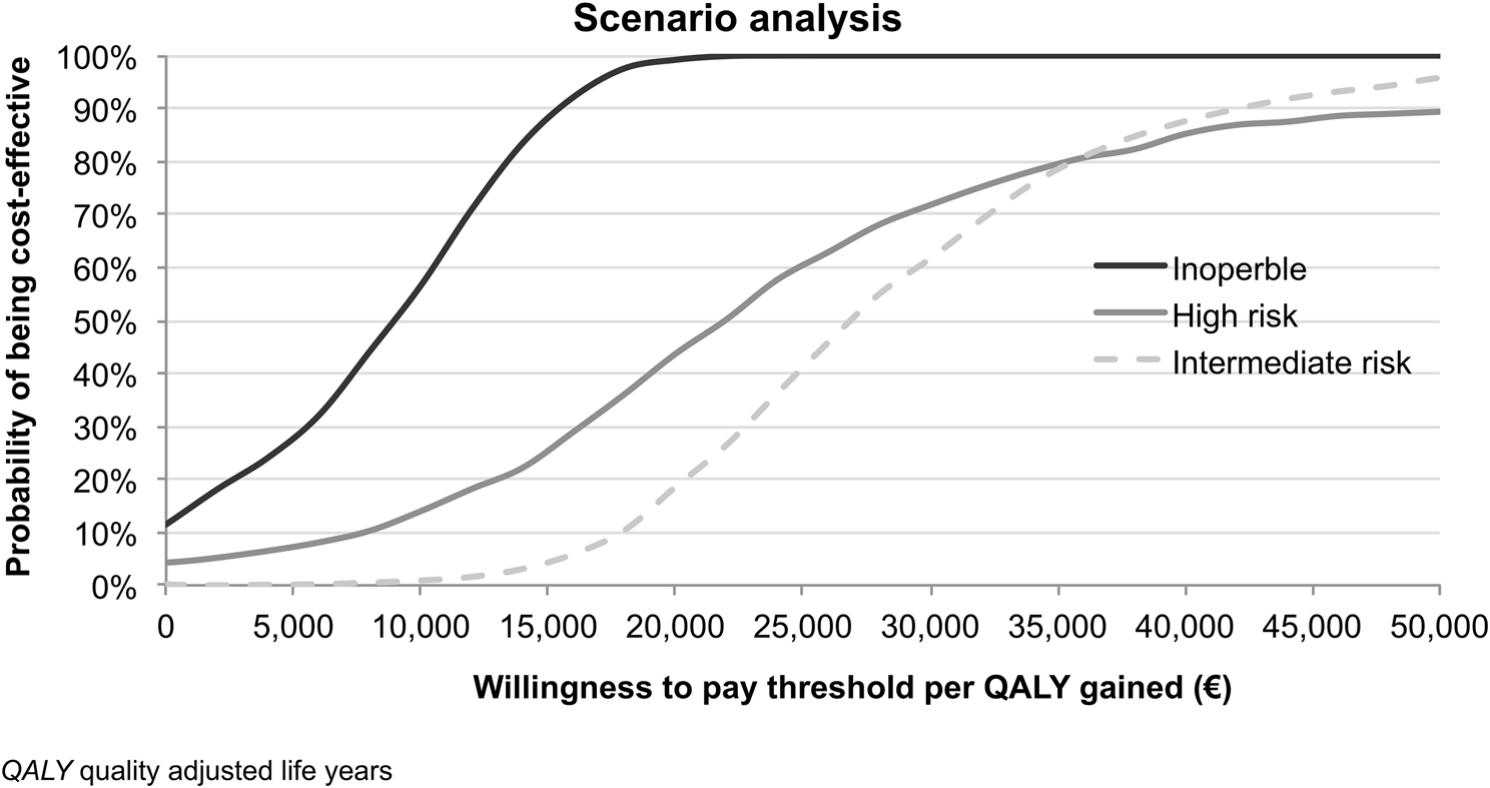
Scenario analysis: cost-effectiveness acceptability curves for TAVI over different risk groups

Also considering CE acceptability curves for the scenario analysis, TAVI resulted in high probability of being cost-effective in all risk groups for a willingness to pay (WTP) of €40,0000. When the WTP threshold is lowered to €25,000 the probability of TAVI being cost-effective was moderate in the intermediate- (40%) and high-risk groups (57.5%). In the inoperable group, TAVI had higher probability of being cost-effective when the WTP is also set to €25,000 (Fig. 6).

## Discussion

The present study provides insight into the value for money of TAVI with the last generation devices over different risk groups in Italy. The results from this study update the existing evidence related to the health economic implication of TAVI in different ways.

To the best of our knowledge, no similar studies have been conducted in the Italian setting and no data are available at present on the health economic evaluations of last generation valves over different risk groups. Moreover, the analyses performed, considering micro-costing data, provides valuable information for health decision and policy makers to understand the economic implication of TAVI in contemporary clinical practice.

Since the first “in man” TAVI procedure in 2002 and the availability of the first generation valve in the market in 2007 [35], several devices became available and there has been an iterative development to reduce the risk of clinical complications and optimize the procedure.

Although TAVI is a minimally invasive procedure, first generation valves showed risk of re-intervention and vascular complications that have been minimized with the last generation valves. These have been improved reducing device height, changing the structure and profile of the device, setting up novel mechanisms to anchor the device and innovating the delivery system to facilitate optimal positioning and deployment of the valve [19, 36].

Accordingly, the effectiveness and safety profile has evolved and indication for the use of TAVI has thus been progressively extended to patients with high-, intermediate- and low-risk in addition to inoperable groups [8, 9].

As a result of device improvement and accumulation of experience in clinical practice, health economic implications of last generation devices could not be easily compared with evidence about first generation valves because of the inner difference of technology compared.

In all the analyses performed in the present study, TAVI resulted in additional LYG and QALY gained at the price of additional costs whose values varied according to the surgical risk group and the analyses performed (i.e., considering tariff or micro-costs, including or not rehabilitation).

In the base-case analysis, when considering intermediate- and high-risk patients, the initial difference of €6000 between the sAVR and TAVI (given respectively by the difference in tariff for DRG 105 and the specific tariff set in the Emilia Romagna region in 2014 corresponding to €30,634) was partially offset over the 15-year time horizon due to reduced rate of follow-up complications with the TAVI minimal invasive approach. Similar offset effects were also observed among patients on medical treatment. In all risk groups, TAVI had a very high probability (from approximately 90–100%) of being cost-effective when considering a WTP of €25,000.

Given the effectiveness of TAVI in reducing risk of events in the follow-up, if the CE analysis from the INHS perspective is performed considering the tariff actually set for interventions on cardiac valves [28], not differentiated for sAVR and TAVI, results will suggest that sAVR is dominated anyway, which would mean that reimbursement would not be able to fully cover real costs of the procedure, most likely constraining centres to inefficiency with possible consequence of the widespread of the procedure.

The results from the present analysis are consistent with those obtained in the few studies available related to the last generation devices. Indeed, four recent studies evaluated the CE of TAVI versus sAVR in intermediate-risk patients [24, 37–39]. Three of them showed TAVI being dominant considering the payer perspective and a lifetime horizon in the US [37], France [24] and Australia [39], while, accounting for the perspective of the National Health System in Canada, Tarride et al. [38] found that TAVI was cost-effective (ICER being 28,154 Canadian dollars/QALY).

To our knowledge, the latter study is also the only available CE evaluation of SAPIEN 3 in high-risk patients and showed incremental costs and incremental QALY resulting from TAVI with ICER being 17,237 Canadian dollars/QALY.

Despite being generally consistent, the results from previous similar studies vary because of the different setting considered and in particular different costs of procedures induced by diverse acquisition costs and tariffs set.

As also shown in the scenario analysis conducted in the present study, despite the offset effects from the minimally invasive approach, procedural costs (and in particular cost differences of the indexed events) have the largest impact on the value for money of TAVI. Furthermore, base-case results were confirmed in the scenario analysis performed, although when considering procedural costs from micro-costing estimates, the probability of TAVI being cost effective was fair to good among intermediate- and high-risk patients, if we consider the lower limits of the range of WTP values conventionally considered in Italy that varied between €25,000 and €40,000 per QALY [34].

Indeed, to strengthen results from the micro-costing scenario, additional analyses were performed varying some of the unit costs obtained from the centres involved in the present study and presented as supplementary data (see Table A1). In brief, as recently published data from other Italian centres [40] outlined significantly higher data for the hourly wage for clinicians and instrumental nurses, the micro-costs for the index intervention of both TAVI and sAVR were re-estimated considering those data. According to these additional analyses and to a micro-costs for TAVI and sAVR intervention equal to €27,257 and €15,387, conclusions were consistent with those shown in the results section with the value of ICERs and ICURs varying by less than €1000 (see Table A1).

In summary, the present study confirmed the CE of TAVI over different risk groups while also considering scenario and sensitivity analysis.

Accordingly, the extensive use of the technology would maximize the manifestation of clinical benefit [9, 41] as well as its economic implications as shown in different field [42]; as the present study also highlighted, there are main points that may impact on the likelihood of current evidence supporting the widespread use of TAVI at a national level to meet estimated requirements: firstly, the actual representativeness of the tariff currently set for procedure performed with advanced technology (as procedural costs largely impact on the overall costs); secondly, the feasibility of using a conventional threshold to assess the value for money of innovative technologies without foreseeing specific criteria for those approaches able to significantly increase life expectancy [43, 44].

With respect to the first point, limited examples going in the direction of negotiating a specific tariff for advanced technology was set previously in Italy, while similar paths are currently emerging in the context of other diseases. In regards to the second point, the recent experience relating to the introduction of innovative cancer therapies opened a large debate about the appropriateness of relying on conventional thresholds without foreseeing exception for particular areas (i.e., end-of-life therapies, cancer, etc.) and that lead some health authorities to develop specific criteria for those circumstances [45, 46]. Although WTP estimated for TAVI met conventional criteria, the theme is whether approaches are able to clearly extend life expectancy and improve QALYs, which should be framed in a different decisional context in which conventional criteria could be relaxed.

Despite the strengths highlighted, the present study has some limitations. Firstly, effectiveness inputs are derived from a clinical study conducted in the US, possibly not matching national context; similarly, for QALY, that again were derived from estimates in the US because of the unavailability of specific data for Italy. Secondly, the scenario analyses considering a micro-costing approach used data from a limited number of centres, possibly failing to adequately represent the national scenario, even if supplementary results using a different source to estimate micro-costs were provided to corroborate results and even provide possible degrees of their variability. Finally, the use of data from pragmatic or observational studies would have worthily completed the picture of current practice at a national level, but similar comprehensive data were not available and should be the focus of future works.

## Conclusions

Despite limitations outlined, to our knowledge this is the first study providing evidence about the CE of TAVI from the INHS perspective over different risk groups and considering last generation devices.

The results of the CE analysis performed show that, considering the INHS perspective, TAVI would be considered highly cost-effective at frequently cited willingness to pay thresholds. Similar conclusions emerged over a range of analyses performed and also modelling a scenario considering micro-costing data. Indeed, the diverse of analyses performed offer the possible range defining the value for money of TAVI and offer important messages to clinicians and decision maker on both the overall value of TAVI, but also on the feasibility of considering the procedure over diverse risk groups, some of which were rarely considered as candidate for TAVI procedure both in view of the limited evidence related to both clinical and economic implications. Further studies may help shed light about CE of TAVI in real-life scenarios, including the impact of the learning curve on health-economic outcomes [47], considering real costs from larger samples and also exploring the perspective of the society to both capture indirect costs and provide insight into the value of the procedure from the patient's point of view [48].

## Supplementary Information

Below is the link to the electronic supplementary material.

Supplementary file1 (DOCX 45 KB) Supplementary data detail inputs used to derive transition probabilities (Table A1) and utility values (Table A2); results from additional analysis performed using different inputs to derive micro-costing estimates for the procedures were also provided (Table A3).
