# Supplementary material for: The cost-effectiveness of transcatheter aortic valve implantation: exploring the Italian National Health System perspective and different patient risk groups
Source: Eur J Health Econ. 2021 May 21;22(9):1349–63. doi: 10.1007/s10198-021-01314-z (PMC8558181; doi:10.1007/s10198-021-01314-z)
Supplement: Supplementary file 1 — Supplementary file1 (DOCX 45 KB) Supplementary data detail inputs used to derive transition probabilities (Table A1) and utility values (Table A2); results from additional analysis performed using different inputs to derive micro-costing estimates for the procedures were also provided (Table A3). [file 10198_2021_1314_MOESM1_ESM.docx]

**Table A1. Details of patients’ distribution from PARTNERS studies used to derive transition probabilities**

|  | **Intermediate risk** | | | | | |
| --- | --- | --- | --- | --- | --- | --- |
|  | **TAVI** | | | **sAVR** | | |
|  | **1-months** | **6-months** | **12-months** | **1-months** | **6-months** | **12-months** |
| NYHA I | 0.010 | 0.039 | 0.041 | 0.035 | 0.048 | 0.051 |
| NYHA II | 0.000 | 0.543 | 0.603 | 0.000 | 0.438 | 0.646 |
| NYHA III | 0.272 | 0.357 | 0.292 | 0.230 | 0.383 | 0.239 |
| NYHA IV | 0.604 | 0.054 | 0.060 | 0.555 | 0.109 | 0.057 |
| Death | 0.114 | 0.006 | 0.004 | 0.180 | 0.021 | 0.006 |
|  | **High risk** | | | | | |
|  | **TAVI** | | | **sAVR** | | |
|  | **1-months** | **6-months** | **12-months** | **1-months** | **6-months** | **12-months** |
| NYHA I | 0.013 | 0.002 | 0.001 | 0.013 | 0.000 | 0.001 |
| NYHA II | 0.000 | 0.487 | 0.627 | 0.000 | 0.208 | 0.468 |
| NYHA III | 0.100 | 0.404 | 0.312 | 0.050 | 0.443 | 0.413 |
| NYHA IV | 0.565 | 0.086 | 0.051 | 0.423 | 0.265 | 0.095 |
| Death | 0.321 | 0.022 | 0.010 | 0.514 | 0.083 | 0.023 |
|  | **Inoperable** | | | | | |
|  | **TAVI** | | | **Medical Treatment** | | |
|  | **1-months** | **6-months** | **12-months** | **1-months** | **6-months** | **12-months** |
| NYHA I | 0.020 | 0.085 | 0.094 | 0.061 | 0.235 | 0.256 |
| NYHA II | 0.000 | 0.339 | 0.444 | 0.000 | 0.029 | 0.019 |
| NYHA III | 0.094 | 0.411 | 0.363 | 0.058 | 0.226 | 0.273 |
| NYHA IV | 0.635 | 0.150 | 0.094 | 0.457 | 0.363 | 0.348 |
| Death | 0.251 | 0.015 | 0.006 | 0.425 | 0.147 | 0.104 |

sAVR= surgical valve replacement; TAVI= transcatheter aortic valve implantation.

**Table A2. Details of main utility values used in the analysis**

|  | **Intermediate risk** | | **High risk** | | **Inoperable** | |
| --- | --- | --- | --- | --- | --- | --- |
|  | **TAVI** | **sAVR** | **TAVI** | **sAVR** | **TAVI** | **Medical treatment** |
| ***1-month*** |  |  |  |  |  |  |
| **NYHA I-IV without stroke** | 0.71 | 0.56 | 0.61 | 0.51 | 0.63 | 0.42 |
| ***6-month*** |  |  |  |  |  |  |
| **NYHA I-IV without stroke** | 0.7 | 0.69 | 0.64 | 0.62 | 0.58 | 0.45 |
| ***1 year^*^*** |  |  |  |  |  |  |
| **NYHA I-IV without stroke** | 0.7 | 0.69 | 0.64 | 0.62 | 0.58 | 0.4 |
| **Average Utility for Stroke** |  | 0.680 | | | | |

*^*^*Utility values for NYHA classes in subsequent year were assumed to remain equal to the 1 year values

sAVR= surgical valve replacement; TAVI= transcatheter aortic valve implantation.

**Table A3. Results of the cost-effectiveness analysis over a 15-year time horizon considering rehabilitation and deriving micro-costing data for the index intervention combining data collected from clinical centres involved in the study and data from Filetti et al. 2017**

|  | **Costs** | **QALY** | **LY** | **Δ costs (€)** | **Δ QALY** | **Δ LY** | **ICUR** | **ICER** |
| --- | --- | --- | --- | --- | --- | --- | --- | --- |
| **Intermediate risk** | | | | | | | | |
| **TAVI** | 33,439 | 4.21 | 6.08 | 6,917 | 0.43 | 0.45 | 16,053 | 15,470 |
| **sAVR** | 26,522 | 3.78 | 5.64 |  |  |  |  |  |
| **High risk** | | | | | | | | |
| **TAVI** | 33,838 | 2.83 | 4.49 | 7,021 | 0.34 | 0.40 | 20,544 | 17,363 |
| **sAVR** | 26,817 | 2.49 | 4.08 |  |  |  |  |  |
| **Inoperable** | | | | | | | | |
| **TAVI** | 31,790 | 1.83 | 3.17 | 9,390 | 1.18 | 1.57 | 7,983 | 5,969 |
| **Medical treatment** | 22,400 | 0.65 | 1.60 |  |  |  |  |  |

ICER=incremental cst-effectiveness ratio; ICUR=incremental cost-utility ratio; LY=life-years; QALY=quality adjusted life years; sAVR= surgical valve replacement; TAVI= transcatheter aortic valve implantation.
